# Generation, Characterization, and Quantitative Bioanalysis of Drug/Anti-drug Antibody Immune Complexes to Facilitate Dedicated *In Vivo* Studies

**DOI:** 10.1007/s11095-019-2661-0

**Published:** 2019-06-28

**Authors:** Eugenia Hoffmann, Gregor Jordan, Matthias Lauer, Philippe Ringler, Eric A. Kusznir, Arne C. Rufer, Sylwia Huber, Anton Jochner, Gerhard Winter, Roland F. Staack

**Affiliations:** 1 Roche Pharma Research and Early Development, Pharmaceutical Sciences, Roche Innovation Center Munich, Penzberg, Germany; 2 Department of Pharmacy, Pharmaceutical Technology & Biopharmaceutics, Ludwig-Maximilians-University, Munich, Germany; 3 Roche Pharma Research and Early Development, Therapeutic Modalities, Roche Innovation Center Basel, Basel, Switzerland; 4 Center for Cellular Imaging and NanoAnalytics (C-CINA), Biozentrum University of Basel, Basel, Switzerland; 5 Roche Pharma Research and Early Development, Therapeutic Modalities, Roche Innovation Center Munich, Penzberg, Germany

**Keywords:** Anti-drug antibodies, bioanalysis, ELISA, immune complex, immunogenicity, therapeutic proteins

## Abstract

**Purpose:**

Immunogenicity against biotherapeutics can lead to the formation of drug/anti-drug-antibody (ADA) immune complexes (ICs) with potential impact on safety and drug pharmacokinetics (PK). This work aimed to generate defined drug/ADA ICs, characterized by quantitative (bio) analytical methods for dedicated determination of IC sizes and IC profile changes in serum to facilitate future *in vivo* studies.

**Methods:**

Defined ICs were generated and extensively characterized with chromatographic, biophysical and imaging methods. Quantification of drug fully complexed with ADAs (drug in ICs) was performed with an acid dissociation ELISA. Sequential coupling of SEC and ELISA enabled the reconstruction of IC patterns and thus analysis of IC species in serum.

**Results:**

Characterization of generated ICs identified cyclic dimers, tetramers, hexamers, and larger ICs of drug and ADA as main IC species. The developed acid dissociation ELISA enabled a total quantification of drug fully complexed with ADAs. Multiplexing of SEC and ELISA allowed unbiased reconstruction of IC oligomeric states in serum.

**Conclusions:**

The developed *in vitro* IC model system has been properly characterized by biophysical and bioanalytical methods. The specificity of the developed methods enable discrimination between different oligomeric states of ICs and can be bench marking for future *in vivo* studies with ICs.

## Introduction

The therapy of a variety of diseases was revolutionized by the development of biotherapeutics. Especially monoclonal antibodies (mAbs) are widely used for the treatment of diseases such as cancer and autoimmune deficiencies.

Technological progress allows the generation of fully human mAbs. Although fully human biotherapeutics are much better tolerated by patients, immune responses against these drugs can occur (1). Immunogenicity against a mAb can be influenced by different extrinsic and intrinsic factors, like manufacturing, formulation, co-medication, immunological status or glycosylation pattern (2,3). Although fully human, immunogenicity can also be triggered by antibody-specific structural amino acid patterns, especially in the complementary determining region (CDR) of Abs (2,4). Consequently, anti-drug antibodies (ADAs) can be generated by the immune system, which bind to the drug and may lead to the formation of immune complexes (ICs) of drug and ADAs. Neutralizing ADAs for example can constrain the binding of the drug to its target. Therefore, formation of ICs can have influence on efficacy and pharmacodynamics of the drug. Additionally, pharmacokinetics (PK) of the drug can be altered by increasing or decreasing drug clearance (5).

To enable dedicated *in vivo* studies investigating the influence of IC formation on drug PK, this paper describes an *in vitro* model for the generation and characterization of defined ICs, as well as the development of bioanalytical methods to quantify drug fully complexed with ADAs to allow IC size-specific PK assessment. In contrast to previous studies with no information about the exact size of formed or found ICs (6,7), our goal was to have a quantitative determination of generated ICs facilitating advanced studies.

**Fig. 1:**
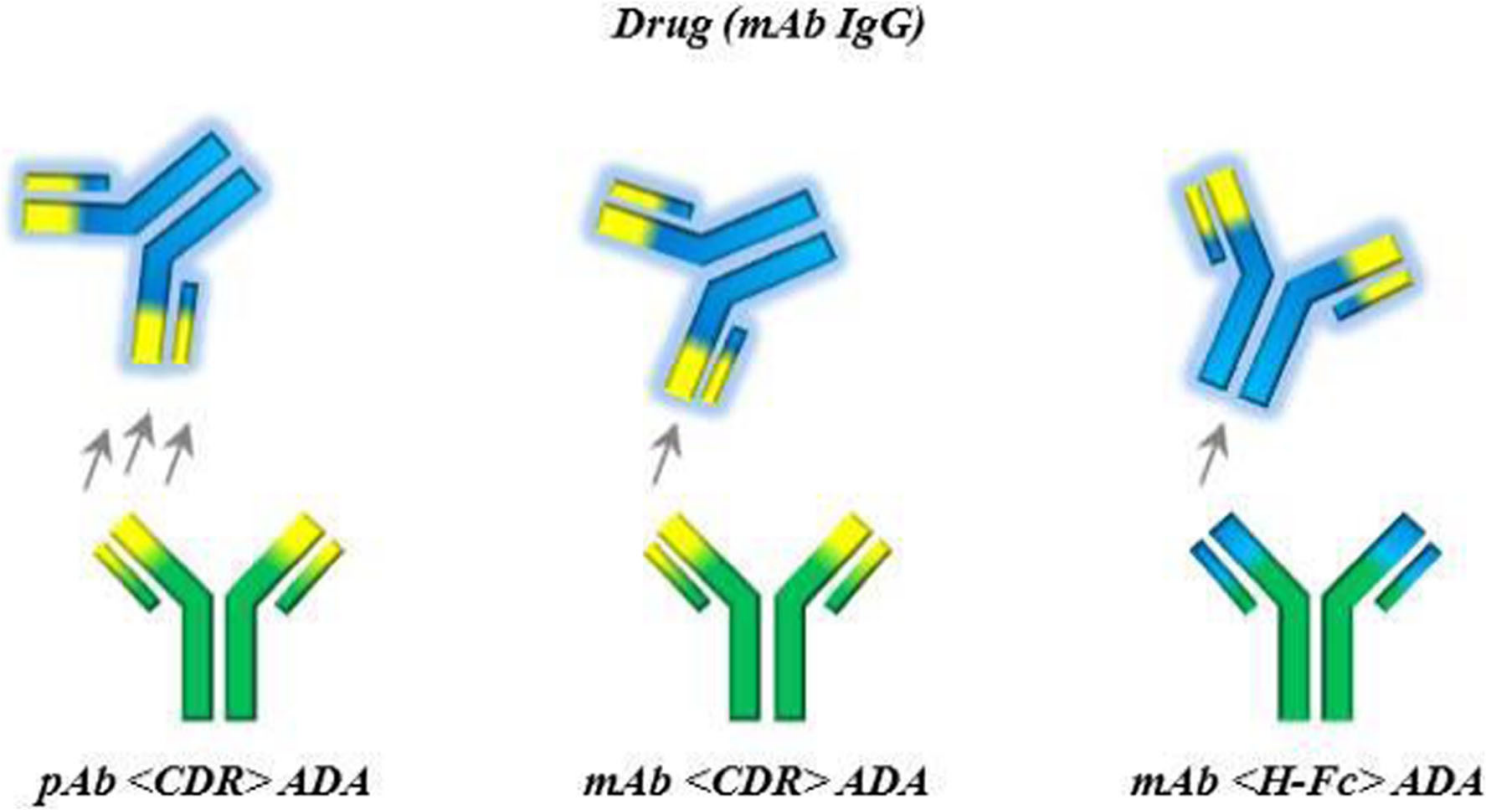
Schematic overview of used ADA surrogates, pAb <CDR> ADA, mAb <CDR> ADA, mAb < H-Fc > ADA and their binding sites to the hIgG_1_ drug.

A human non-binding IgG_1_ (PGLALA) mAb was chosen as drug model (8–11). As ADA surrogate a polyclonal Ab (pAb) against the CDR (pAb <CDR> ADA) of the drug was used. Additionally, a mAb against the CDR and the Fc region of the drug were used as ADA surrogates for a more detailed understanding of ADA clonality and epitope on IC formation (see Fig. 1 and discussion).

## Materials and Methods

### Compounds

As drug surrogate a human monoclonal IgG_1_ without target specificity was used. Three different Abs were used as ADA surrogates: a mouse-derived monoclonal IgG against the Fc portion of human IgG, a mouse-derived monoclonal IgG against the CDR of the drug, and a polyclonal rabbit-derived IgG against the CDR of the drug. All mentioned Abs, as part of the ICs or as capture and detection reagents were produced in house (Roche Diagnostics GmbH, Penzberg, Germany). Biotin (Bi)- and digoxigenin (Dig)-labeling of appropriate Abs was performed in house.

### Reagents

Bovine serum albumin (BSA), Roche universal buffer for ELISA (RUB), phosphate-buffered saline (PBS), and Tween20 (10% (*w*/*v*)) were obtained from Roche Diagnostics GmbH (Mannhein, Germany). Rat serum (female, Wistar) was from Charles River Laboratories (Sulzfeld, Germany). Ethanol, 30% H_2_O_2_, and Tris(hydroxymethyl)aminomethane (Tris/HCl) were ordered from Merck KGaA (Darmstadt, Germany). Glycine and 3-(4-Hydroxyphenyl) propionic acid (HPPA) were obtained from Serva Electrophoresis GmbH (Heidelberg, Germany) and Sigma Aldrich (St. Louis, MO, USA), respectively.

### Generation of Immune Complexes

Generation of ICs was performed by mixing the drug surrogate with one of the ADA surrogates in PBS or rat serum. Different ratios and different concentrations were tested. For analysis of IC formation, Ab mixtures were incubated at room temperature (r.t.) for 1 h on a shaker with 500 rounds per minute (rpm). Stability and kinetic studies were performed at 37°C for serval days while shaking.

### Size Exclusion Chromatography (SEC)

For chromatographic analysis a Dionax UltiMate 3000 system from Thermo Fisher Scientific GmbH (Dreieich, Germany) was used (UV detector MWD-3000, auto sampler, automated fraction collector) with a Waters XBridge Protein BEH SEC Guard Column, 450 Å, 3.5 μm, 7.8 mm X 30 mm and XBridge Protein BEH SEC Column, 450 Å, 3.5 μm, 7.8 mm X 300 mm (Milford, MA, USA). As SEC protein standard, BEH450 SEC Protein Standard Mix from Waters was used (Milford, MA, USA). 20 μl of centrifuged buffer or serum sample were injected for SEC analysis. PBS with 5% ethanol (*v/v*) was used as running buffer with a flow rate of 0.5 ml/min. Online UV detection of buffer samples was performed at 280 nm. Fractions were collected every 30 s into 96-deep well V-shape plates (Masterblock, 0.5 ml, greiner bio-one, Kremsmünster, Austria). To avoid aggregation or unspecific binding of collected protein, high concentrated BSA buffer (10.5% BSA (*w*/*v*) in PBS + 5% ethanol (*v/v*)) was pre-added to achieve a final concentration of 0.5% BSA.

### SEC-Multiple Angle Light Scattering (SEC-MALS)

50 μl of single Abs and ICs (1:1.5 ratio of drug to ADA) were prepared in PBS with a total concentration of 1 mg/ml. For SEC conditions, see above. As MALS detector, a Wyatt miniDawnTREOS/QELS- and Optilab rEX detector was used (Wyatt technology, Santa Barbara, CA, USA). Evaluation was performed with Astra 6.1 from Wyatt.

### Analytical Ultracentrifugation (AUC)

Sedimentation velocity experiments were performed at 20,000 rpm in an An-60 Ti rotor on a XLI instrument (Beckman Coulter, CA, USA) at 20°C with SedVel60k (12 mm optical path length, SpinAnalytical, ME, USA) centerpieces. The sedimentation process was monitored with absorbance detection at 280 nm.

Concentrated protein solutions containing ICs of drug and pAb <CDR> ADA were prepared in PBS (1:1.5). IC species (named “Dimer”, “Tetramer”, and “Hexamer” of drug and ADA) were separated by SEC with PBS as running buffer prior to AUC analysis. Collected fractions (peak collection, without BSA as additive) with specific IC species were adjusted to the required concentrations and stored at −20°C till AUC measurement. ICs fractions were analyzed by AUC at total protein concentrations of 0.2 mg/ml.

Sedimentation data were analyzed with Sedfit (open access software, www.analyticalultracentrifugation.com) using continuous sedimentation coefficient distributions c(s) (12,13). Sedimentation coefficient distributions were visualized with GUSSI (14).

### Negative Staining Transmission Electron Microscopy (NS-TEM)

Highly concentrated solutions of ICs (2 mg/ml drug +3 mg/ml ADA) were prepared in PBS. The IC species were separated via SEC with PBS as running buffer. Collected fractions (peak collection, without BSA as additive) with specific IC species (mainly dimers and tetramers) were stored at −20°C. Freshly thawed samples/fractions were diluted in PBS and 4 μl were adsorbed for 60 s to glow-discharged parlodion carbon-coated copper grids. The grids were then blotted, washed with three drops of double-distilled water, incubated with 2 μl of Tobacco Mosaic Virus solution (TMV; kindly supplied by Ruben Diaz-Avalos, New York Structural Biology Center, USA), further washed with two drops of water and finally negatively stained with two drops of 2% uranyl acetate (pH 4.3) solution. Samples were imaged at a nominal magnification of 52,000x using a Tecnai12 transmission electron microscope (FEI, Eindhoven, Netherlands) operating at 120 kV. Electron micrographs were recorded on a 4000 by 4000 pixel charge-coupled device camera (F416, Tietz Video and Image Processing System, Gauting, Germany) yielding a final pixel size of 0.25 nm on the specimen level. Reference-free alignment was performed on manually selected fibril segments from recorded images using the EMAN2 image processing package (15).

### Acid Dissociation ELISA: Quantification of Drug Fully Complexed with ADAs

Calibration standards were prepared with monomeric drug ranking from 375 ng/ml to 0.51 ng/ml, using PBS and 0.5% BSA with 1% rat serum. Quality control (QC) samples of the drug covering the whole calibration range (high concentration QC (H-QC, 30,000 ng/ml drug), mid-concentration QC (M-QC, 15,000 ng/ml drug), and low concentration QC (L-QC, 750 ng/ml drug)) were prepared in 100% rat serum and diluted 100-fold in PBS and 0.5% BSA to control assay performance and exclude technical issues. Defined concentrations of drug in complex with the ADA surrogate were prepared in 100% rat serum and incubated for at least 1 h at r.t (H-IC-QC:30,000 ng/ml drug +45,000 ng/ml ADA; M-IC-QC: 15,000 ng/ml drug +22,500 ng/ml ADA; L-IC-QC: 750 ng/ml drug +1125 ng/ml ADA). After incubation, IC-QCs were diluted 100-fold in PBS and 0.5% BSA. The sample to be quantified (in 100% serum) was analyzed at three dilutions (e.g. 100-, 1000-, and 3000-fold). The final serum concentration in diluted samples was 1%. In case of samples in buffer, no serum was added to calibrators, QCs or dilutions. 20 μl of calibration standards, QCs, IC-QCs, and diluted samples were mixed with 100 μl of 0.1 M glycine-HCl, pH 2 in V-shaped 96-well plates (Masterblock, 0.5 ml, greiner bio-one, Kremsmünster, Austria) and incubated 30 min at r.t. on a microplate shaker at 500 rpm. For capture and detection of the drug (in the presence of CDR specific ADAs) a solution of 3 μg/ml Bi-labeled mAb against the PGLALA mutation of the drug and 3 μg/ml Dig-labeled mAb against human IgG-Fc was prepared in RUB. For samples containing drug Fc-specific ADAs, 3 μg/ml Bi-labeled and Dig-labeled mAbs against the CDR of the drug were used. 150 μl of the capture/detection solution were added to the acidic samples and immediately neutralized by the addition of 30 μl 0.5 M Tris/HCl, pH 8.5. After further 30 min incubation, 100 μl of the sample (in duplicates) were transferred on a white streptavidin-coated 96-well microtiter plate (Microcoat GmbH, Bernried, Germany) and incubated for at least 1 h at r.t. on a microplate shaker at 500 rpm. Wells were washed three times with 300 μl PBS + 0.05% Tween20 using a microplate washer (EL405 select, BioTek instruments, Inc., VT, USA). 100 μl of 20 mU/ml horseradish peroxidase (HRP)-conjugated anti-Dig Fab fragments (Roche Diagnostics GmbH, Mannheim, Germany) in RUB were added in each well and incubated for another 1 h under already described conditions. After washing, 100 μl of HPPA solution (3.3 mg/ml HPPA and 0.005% H_2_O_2_ in 0.1 M Tris/HCl, pH 8.5) were added in each well and the plate was shaken for 10 min. Fluorescence read out (excitation wavelength: 320 nm, emission wavelength: 405 nm) was performed with a Tecan Infinite F 200 plate reader (Männedorf, Switzerland). A nonlinear four-parameter curve fitting (Wiemer-Rodbard) was used for the calibration curve allowing a recalculation of sample concentrations.

### SEC of Serum Samples: Reconstruction of IC Patterns in Serum

20 μl of centrifuged sample (in buffer or serum) containing drug fully complexed with ADAs were separated by SEC and fractions were collected (see SEC). As described above, BSA was pre-added in the collection tubes to avoid unspecific binding or aggregation. Calibration standards (375 ng/ml to 0.51 ng/ml) were prepared with monomeric drug, using PBS, 5% ethanol and 0.5% BSA (assay buffer). QCs (H-QC:300 ng/ml drug; M-QC: 150 ng/ml drug; L-QC:7.5 ng/ml drug) and IC-QCs (H-IC-QC:300 ng/ml drug +450 ng/ml ADA; M-IC-QC: 150 ng/ml drug +225 ng/ml ADA; L-IC-QC:7.5 ng/ml drug +11.25 ng/ml ADA) were prepared in assay buffer. Collected fractions were diluted with assay buffer, when necessary. 20 μl of calibration standards, QCs, IC-QCs, and fractions were mixed with 100 μl of 0.1 M glycine-HCl, pH 2 in a 96-well plate. For detailed procedure, see Acid dissociation ELISA. Reconstruction of the IC pattern was performed by plotting the concentration of the drug determined by acid dissociation ELISA in a certain fraction against the elution time of this fraction.

## Results

### SEC: Detection of ICs Via UV

**Fig. 2:**
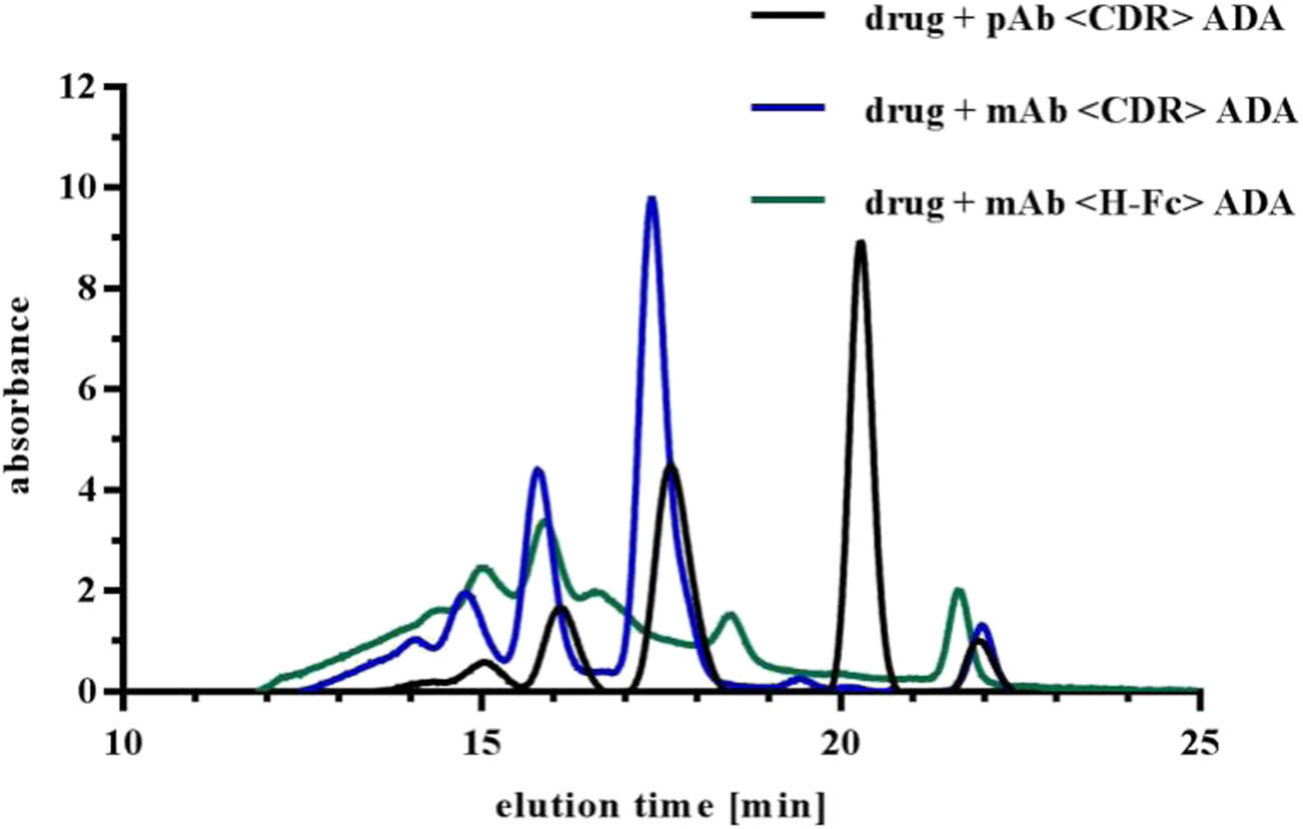
Influence of ADA property on IC pattern. The drug was incubated with pAb <CDR> ADA, mAb <CDR> ADA, and mAb < H-Fc > ADA (1:1.5), respectively. After incubation, ICs were separated via SEC and absorbance was monitored at 280 nm. Elution of monomeric Abs is anticipated at approximately 21.5 to 22 min.

**Fig. 3:**
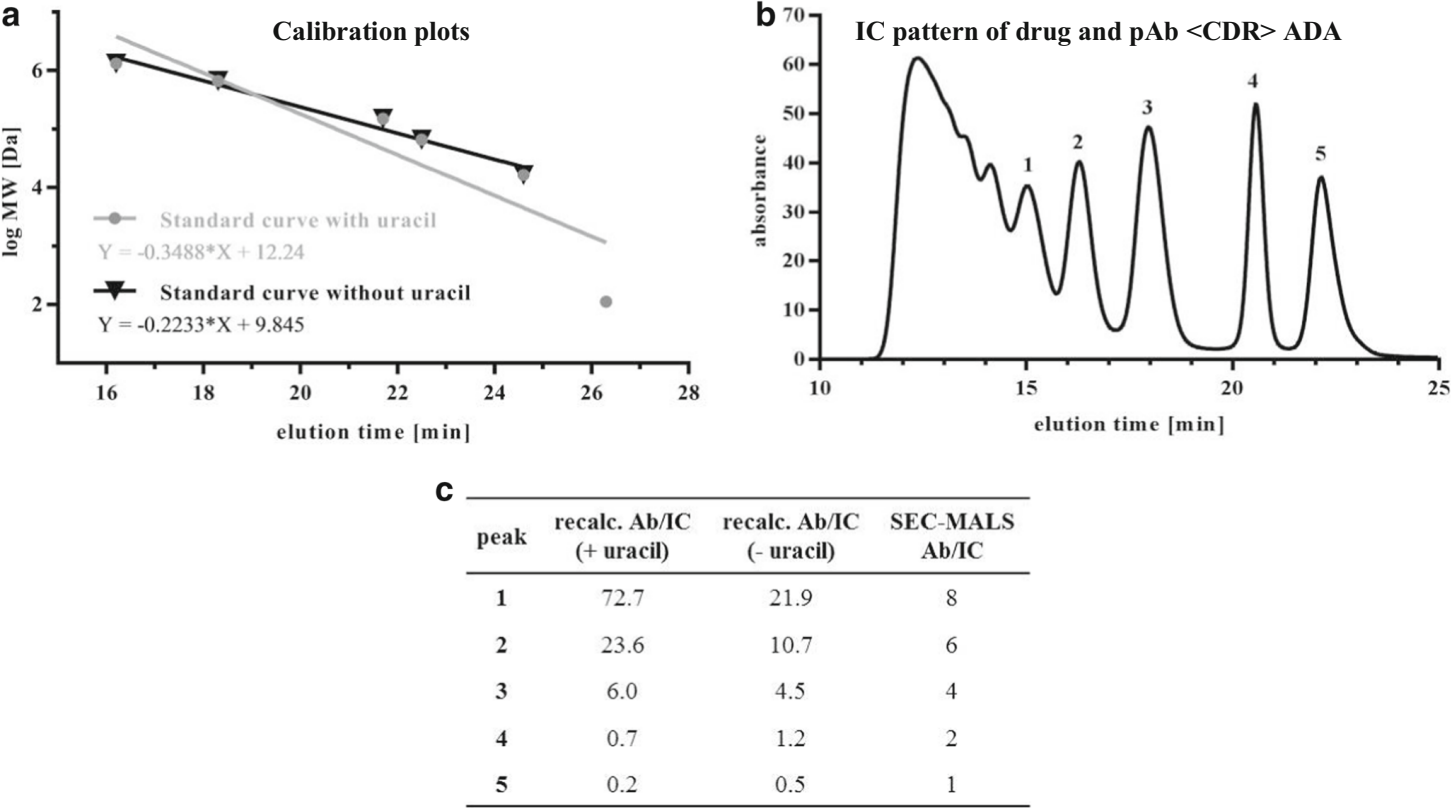
Molecular weight calibration of SEC column. (**a)** Calibration plots of commercial SEC protein standard mix with and without consideration of uracil. (**b)** Exemplary IC mix of drug and pAb <CDR> ADA in buffer separated by SEC and monitored at 280 nm. Single IC species are marked with numbers. (**c)** Summarized recalculation of Ab per complex (Ab/IC) of separated IC species by SEC protein standard mix with and without uracil. Actual numbers (i.e. MWs of separated IC species) were determined by SEC-MALS.

For separation and analysis of formed IC species of drug and ADA in buffer, SEC was utilized as standard analytical method. The online detection of IC separation was performed at 280 nm. Figure 2 shows examples of SEC-separated ICs with different ADA surrogates. To allow an estimation of formed IC sizes, the SEC column was calibrated with a commercial SEC protein standard mix containing molecules from 1320 kDa to 112 Da. The standard mix was separated via SEC and the elution times were plotted against the (logarithmic) molecular weight (MW) of protein standard compounds (Fig. 3a). Additionally, uracil, the smallest component of the standard mix, was excluded from the plot to study the effect on recalculation (see discussion).

An IC mixture of drug and pAb <CDR> ADA was separated via SEC (Fig. 3b) and the MWs of separated peaks were recalculated with the protein standard plots (with and without inclusion of uracil). Figure 3c illustrates the different recalculated values for the MW of studied IC species. Recalculation with the plot excluding uracil resulted in lower values for earlier elution times and higher values for later elution times compared to the recalculation including uracil. The actual MWs of the IC species were determined by SEC-MALS (see below) indicating a higher accuracy of MW recalculation when uracil was not considered (see below and discussion).

### Determination of MWs of Generated Immune Complex Species by SEC-MALS

To overcome the limitations of MW determination via a protein standard mix (see above and discussion), MWs of ICs were determined by SEC-MALS. The drug was mixed with ADAs and formed ICs were separated by SEC followed by online MALS detection. The average MWs and Ab contents of defined peaks were determined and summarized in Table I. The values for the theoretical MWs in Table I base on the assumption of 149 kDa per Ab monomer. For ICs formed with the drug and pAb <CDR> ADA four IC species could be identified corresponding to an octamer, hexamer, tetramer, and dimer of drug and ADA. Furthermore, high molecular weight (HMW) species (defined as bigger than octamers) could be visualized but not further specified because of technical limitations.

**Table I:** Found MWs of Abs and IC species analyzed by SEC-MALS

Sample	Peak	found MW [kDa]	theo. MW [kDa]^1^	Ab/complex
drug + pAb <CDR> ADA	1	n.a.^2^	–	HMW
	2	1254	1192	8
	3	866	894	6
	4	566	596	4
	5	281	298	2
drug + mAb <CDR> ADA	1	n.a.^2^	–	HMW
	2	1120	1192	8
	3	833	894	6
	4	562	596	4
drug + mAb < H-Fc > ADA	1	n.a.^2^	–	HMW
	2	532	447–596	3–4
	3	337	298	2

^1^The theoretical MW was calculated using 149 kDa for an Ab monomer

^2^n.a.: not analyzable due to technical limitations

Additionally, ICs of drug and mAb <CDR> ADA as well as drug and mAb < H-Fc > ADA were analyzed by SEC-MALS (Table I). It could be shown that for tested concentrations ICs with drug and mAb or pAb <CDR> ADAs resulted in same species apart from dimers, which were exclusively found in ICs with pAb <CDR> ADA. ICs of drug and mAb < H-Fc > ADA formed two IC species which could be identified via SEC-MALS: dimers and tetramers of drug and ADA.

### Confirmation of Immune Complex MWs by AUC

**Fig. 4:**
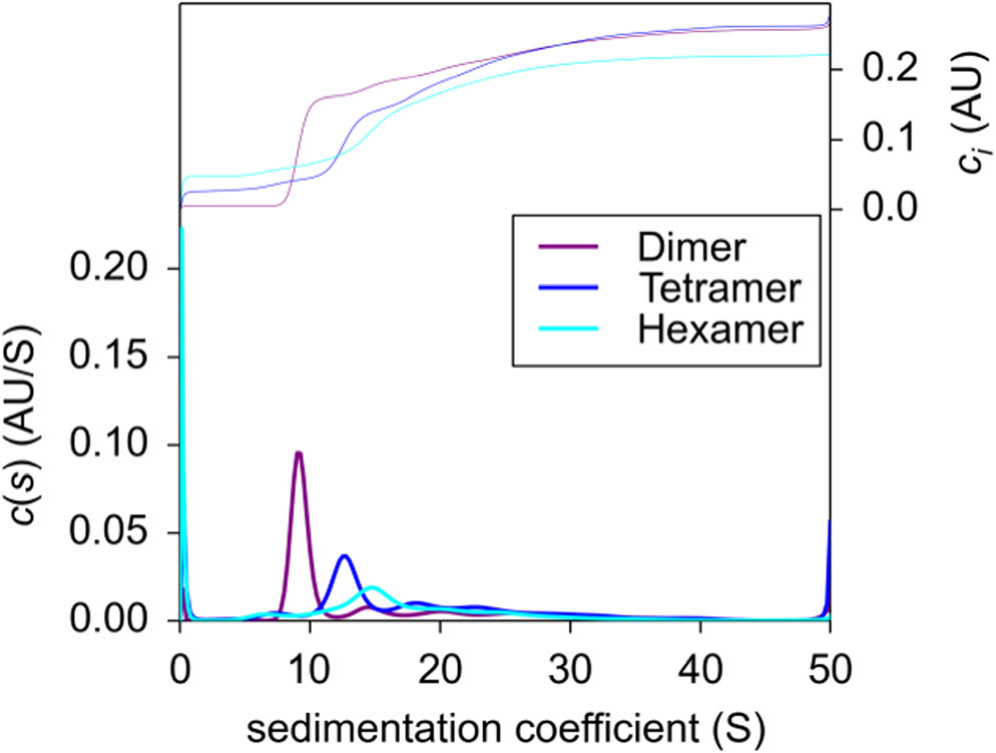
Sedimentation coefficient distributions c(s) for dimer, tetramer and hexamer samples. Defined IC species were separated by SEC and fractionated (named dimer, tetramer, and hexamer fraction). Collected species were analyzed by AUC. The upper curves show the integrated distributions (total loading signal at 280 nm).

For AUC analysis, ICs of drug and pAb <CDR> ADA were generated and the dimer, tetramer and hexamer species (as identified by SEC-MALS) were separated and fractionated by SEC. Separate analysis of the sedimentation coefficient distributions c(s) of collected fractions showed that the three fractions contained protein species with signal weighted average sedimentation coefficients ranging from sw = 6.5 S, which corresponds to an antibody monomer, to species with sw > 50 S, which indicates formation of large ICs (Fig. 4). Notably, each fraction contained a dominant main species with sedimentation coefficients of 9.3 S, 12.8 S and 14.5 S for the fraction designated as Dimer, Tetramer and Hexamer, respectively (Fig. 4). Peak integration showed that these main species accounted for approximately one third to one half of the total loading signals of the individual fractions (Table II). The MW calculated from the sedimentation coefficients of the main species in each of the oligomer fractions agreed within 10% with the theoretical MW of the designated species. Thus, the AUC analysis confirms the presence of dimer, tetramer and hexamer species in collected fractions as identified by SEC-MALS (even after extensive sample treatment including SEC, fractionation, concentration, freeze/thaw cycles, etc., see also Discussion).

**Table II:** AUC data of SEC-separated IC species. ICs of drug and pAb <CDR> ADA were separated by SEC and each collected fraction was analyzed by AUC

Sample/collected SEC peak	sw [S]	Frictional ratio f/f0	calc. MW [kDa]	theo. MW. [kDa]^1^	Species	relative species abundance [%]^2^
“Dimer”	9.3	1.70	297	298	Dimer	59
“Tetramer”	12.8	1.87	557	596	Tetramer	38
“Hexamer”	14.5	2.10	802	894	Hexamer	44

^1^The theoretical MW was calculated using 149 kDa for an Ab monomer

^2^Relative species abundance (area %) were calculated with Sedfit by integration of c(s) peaks

### Confirmation of Immune Complex Molecular Structure by NS-TEM

**Fig. 5:**
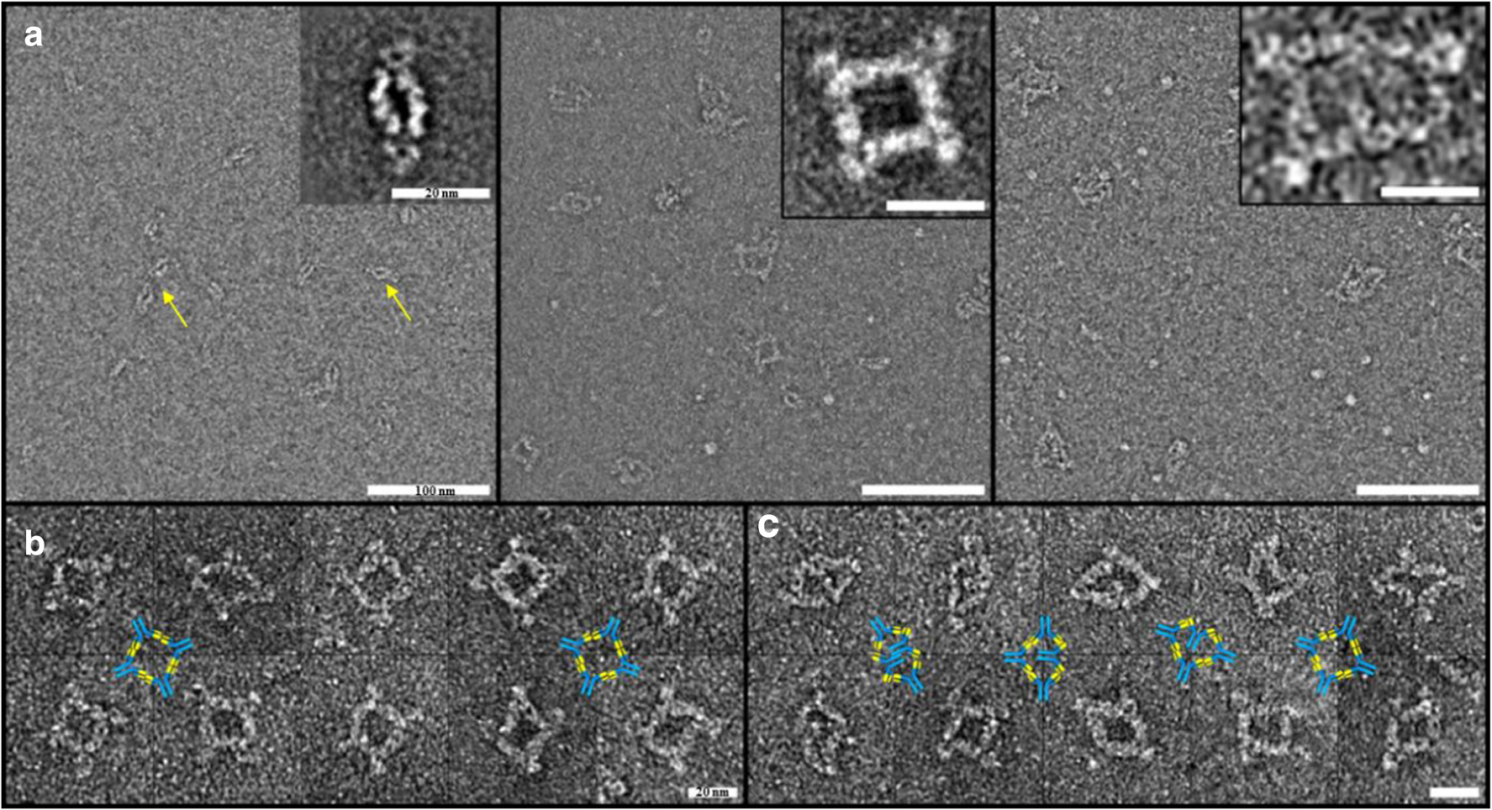
NS-TEM images of ICs. ICs were generated, separated by SEC and defined fractions were collected containing IC species to be analyzed via NS-TEM. (**a)** ICs of drug and pAb <CDR> ADA. The raw micrographs demonstrate a section of the found ICs in analyzed fractions, e.g. dimers in the left, tetramers in the middle, and hexamers in right picture (100 nm scale). The inserts (20 nm scale) represent 2D average images of various particles. (**b)** Found tetrameric structures of ICs of drug and mAb <CDR> ADA. The cartoon illustrates the predominantly found structural arrangement with the IgG-Fc portions facing outwards of the ring structure (20 nm scale). (**c)** Found tetrameric structures of ICs of drug and pAb <CDR> ADA. The cartoons illustrate the different structures with the IgG-Fc portions facing inwards and outwards of the ring (20 nm scale).

A further orthogonal method to determine the mass and structure of generated ICs was NS-TEM. ICs were formed with drug and pAb <CDR> ADA and separated by SEC. Collected fractions contained defined species, like dimers or tetramers (see SEC-MALS and AUC). The raw micrographs of the analyzed fractions (Fig. 5a) qualitatively reflect the size trends detailed in Table II, confirming the presence of dimeric, tetrameric, and hexameric ICs. The complexes are structurally disordered, preferentially forming rings of IgGs that become more disordered the larger they get. The coiled rings reflect a multitude of different chain motifs as different epitopes result in many diverse elementary drug_Fab_-ADA_Fab_ ring segments with various angular orientations (Fig. 5a, c). The IgG-Fc portions in ICs of drug and pAb <CDR> ADAs can therefore lie outside as well as inside the ring (Fig. 5c).

In contrast, tetrameric ICs with mAb <CDR> ADAs are more uniform due to homogenously structured elementary Fab-Fab building motifs (Fig. 5b). Therefore, all Fc portions seem to face outwards of the ring. No dimeric complexes were found under studied conditions (in accordance with UV-SEC and SEC-MALS).

### Generation of Defined Immune Complexes

**Fig. 6:**
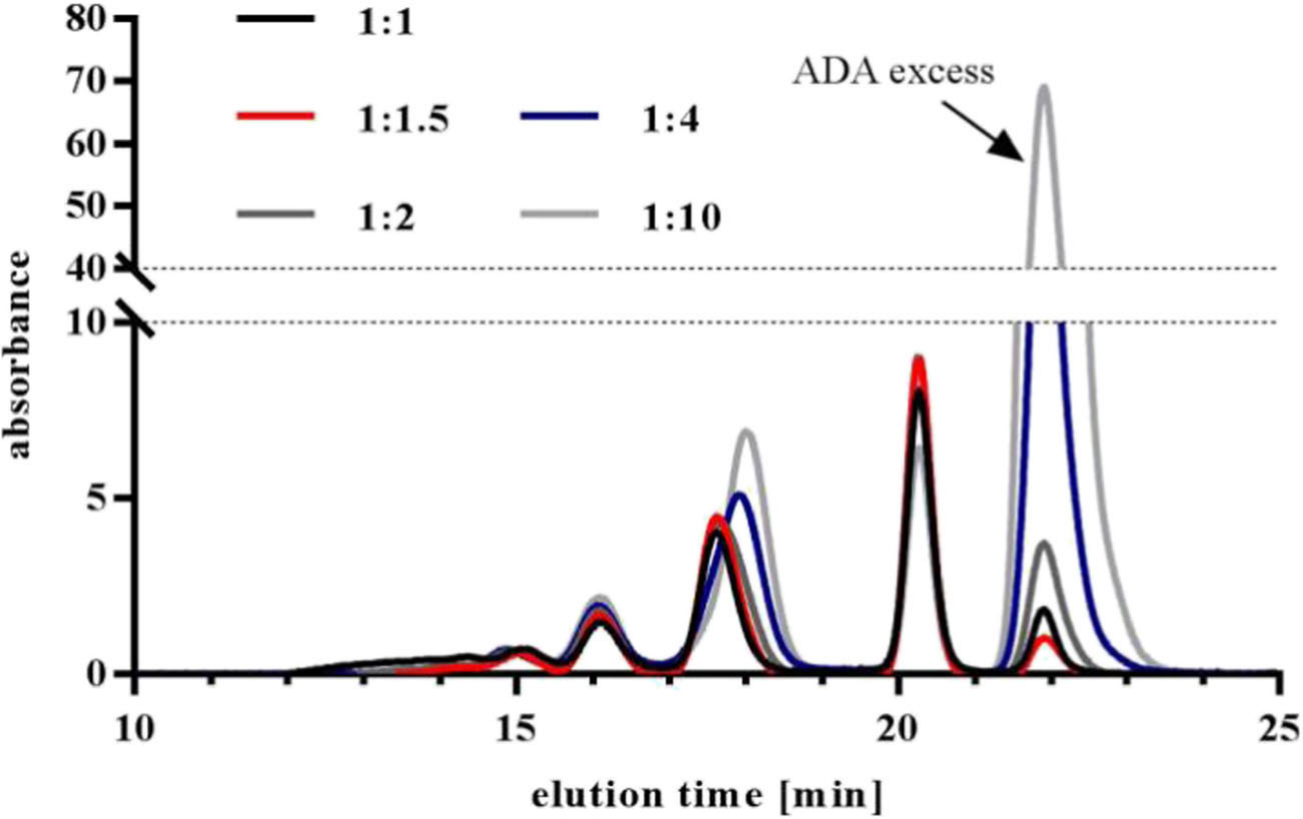
Influence of drug to ADA ratio on IC pattern.100 μg/ml drug were incubated with increasing concentrations of pAb <CDR> ADA, starting with equal amounts up to ten-fold excess of ADA. The peak at 21.8–22 min shows the increasing amount of unbound ADA.

**Fig. 7:**
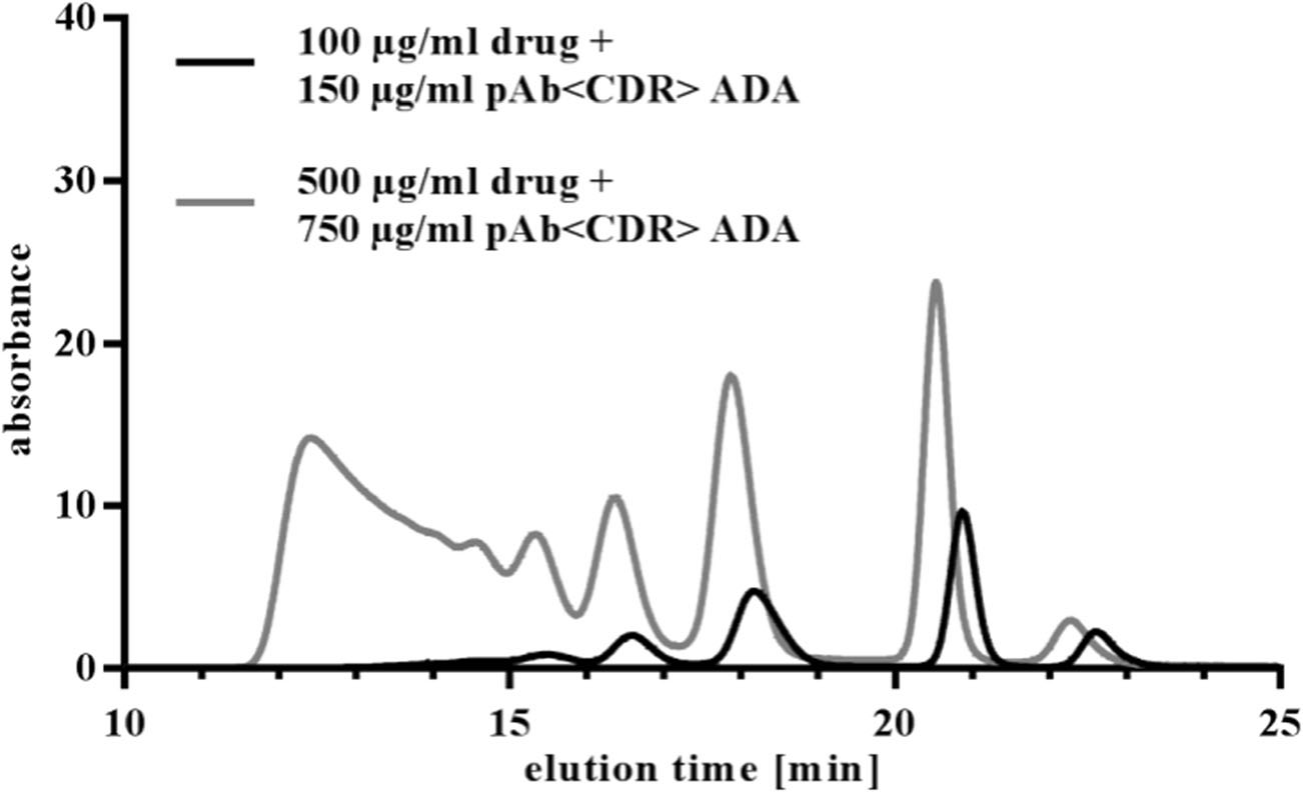
Comparing UV-SEC chromatograms of drug entirely complexed with pAb <CDR> ADA at lower (100 μg/ml) and higher (500 μg/ml) concentrations of drug and pAb <CDR> ADA (1:1.5).

With regard to future *in vivo* studies with preformed ICs, we decided to generate ICs with fully complexed drug. Furthermore, excess of ADA should be as low as possible. Different drug:ADA ratios were tested with increasing ADA concentrations. The effect on the overall IC pattern and the excess monomeric fraction was visualized by UV-SEC. The absence of monomeric drug was tested by two different ways: with fluorescently labeled drug (data not shown) and with an ELISA-based method (see below). Increasing ADA concentrations had little effect on the IC pattern always resulting in comparable SEC chromatograms (Fig. 6). A drug to ADA ratio of 1:1.5 fulfilled all set criteria (no monomeric drug, little ADA excess) and was used as standard ratio for all tested ICs. Furthermore, it was demonstrated that rising concentrations of drug and ADA (but constant ratio) led to increasing HMW content but no changes in the dimer, tetramer, and hexamer fraction (Fig. 7).

Equilibrium, stability, and reproducibility of generated ICs were tested utilizing established UV-SEC. To evaluate the minimum time needed for a stable equilibrium between the IC species of drug and pAb <CDR> ADAs kinetics were performed by analyzing the IC mixture at defined time points (data not shown). It was demonstrated that equilibrium was reached within 1 h at the used concentrations (100 μg/ml drug +150 μg/ml pAb <CDR> ADA) at r.t., which was reasoned from no further changes in SEC pattern.

**Fig. 8:**
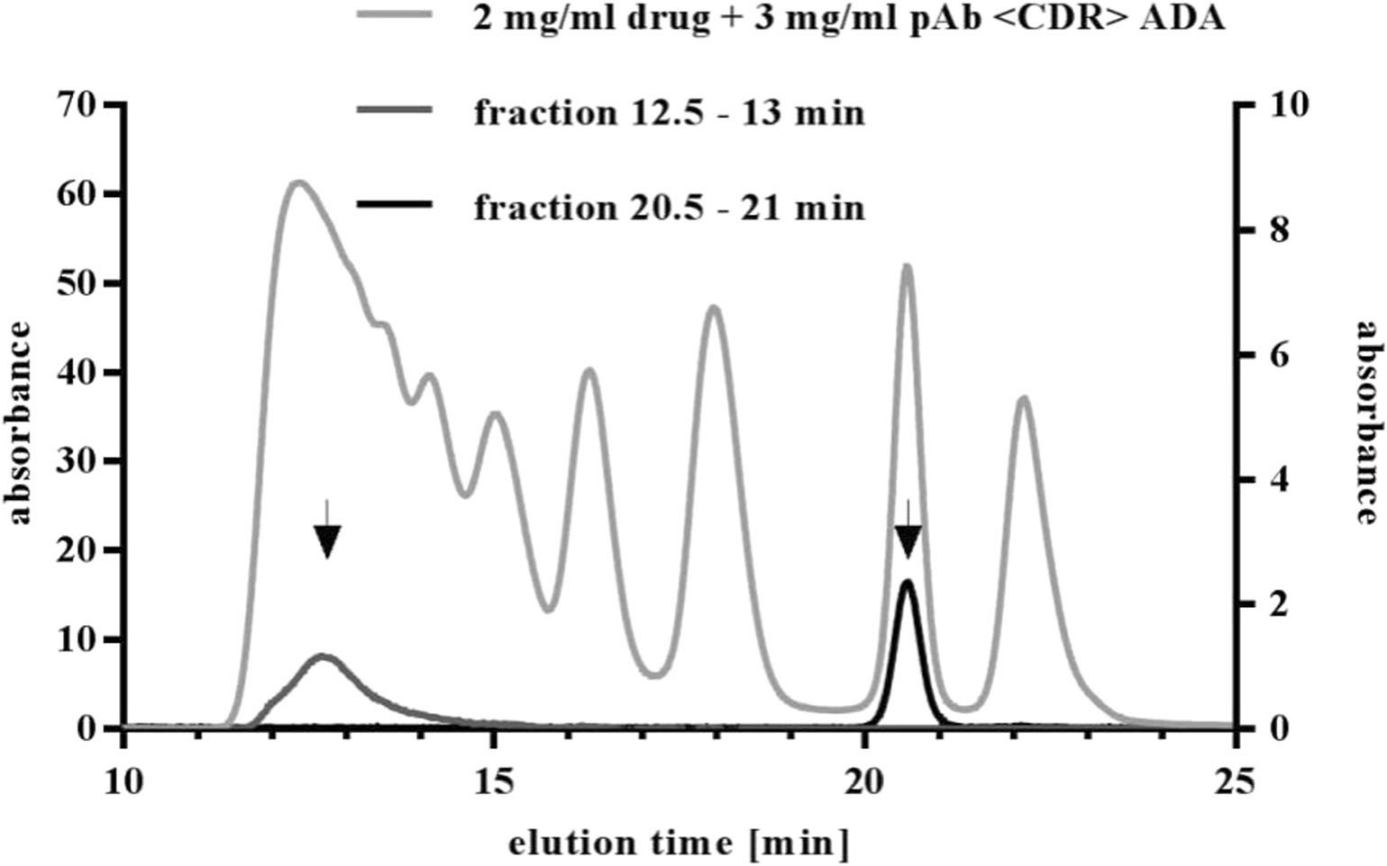
Stability of generated IC species. ICs of drug and pAb <CDR> ADA were separated by SEC and fractions were collected. Selected fractions (marked with arrows) were reanalyzed by UV-SEC, eluting in the expected time frame of 12.5–13 min and 20.5–21 min. No further peaks appear in reinjected samples.

Stability of generated ICs was tested in two different ways: dilution/reinjection and temperature stress. Generated ICs were separated by SEC and two fractions were collected at defined time points (12.5–13 min and 20.5–21 min) representing a HMW and a dimeric IC species, respectively. Collected fractions were reinjected into the SEC and elution times were compared with the previous separation (Fig. 8, expected elution time marked with arrows). Reinjected fractions showed no shift in elution times or appearance of further peaks, indicating high dilution stability of generated ICs independent of studied IC species.

**Fig. 9:**
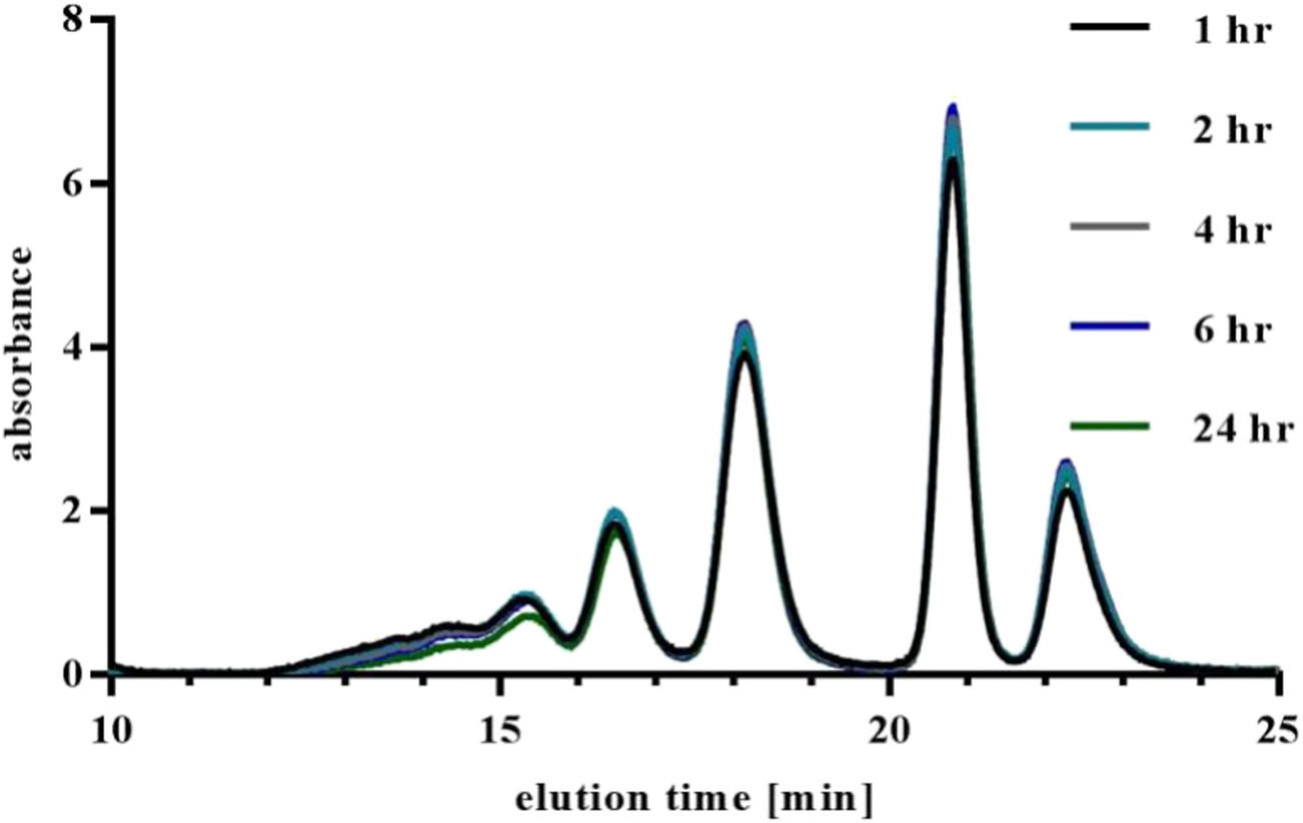
Stability of ICs at 37°C. Generated ICs of drug and pAb <CDR> ADA were incubated for 24 h at 37°C in PBS and the peak pattern was analyzed at defined time points (1 h, 2 h, 4 h, 6 h, and 24 h after IC generation) by UV-SEC.

The temperature stability of generated ICs was tested by incubating the ICs for 24 h at 37°C in PBS (without further additives or stabilizers) and frequent SEC analysis of the ICs (Fig. 9). No changes in IC pattern were detected during selected time period. The coefficient of variation (CV) of the area under the curve was 13.5%. It can be concluded that no rearrangement or significant aggregation of ICs occurred. Furthermore, it was shown that ICs incubated at r.t. or 37°C had the same pattern (Fig. 2, Fig. 9).

**Fig. 10:**
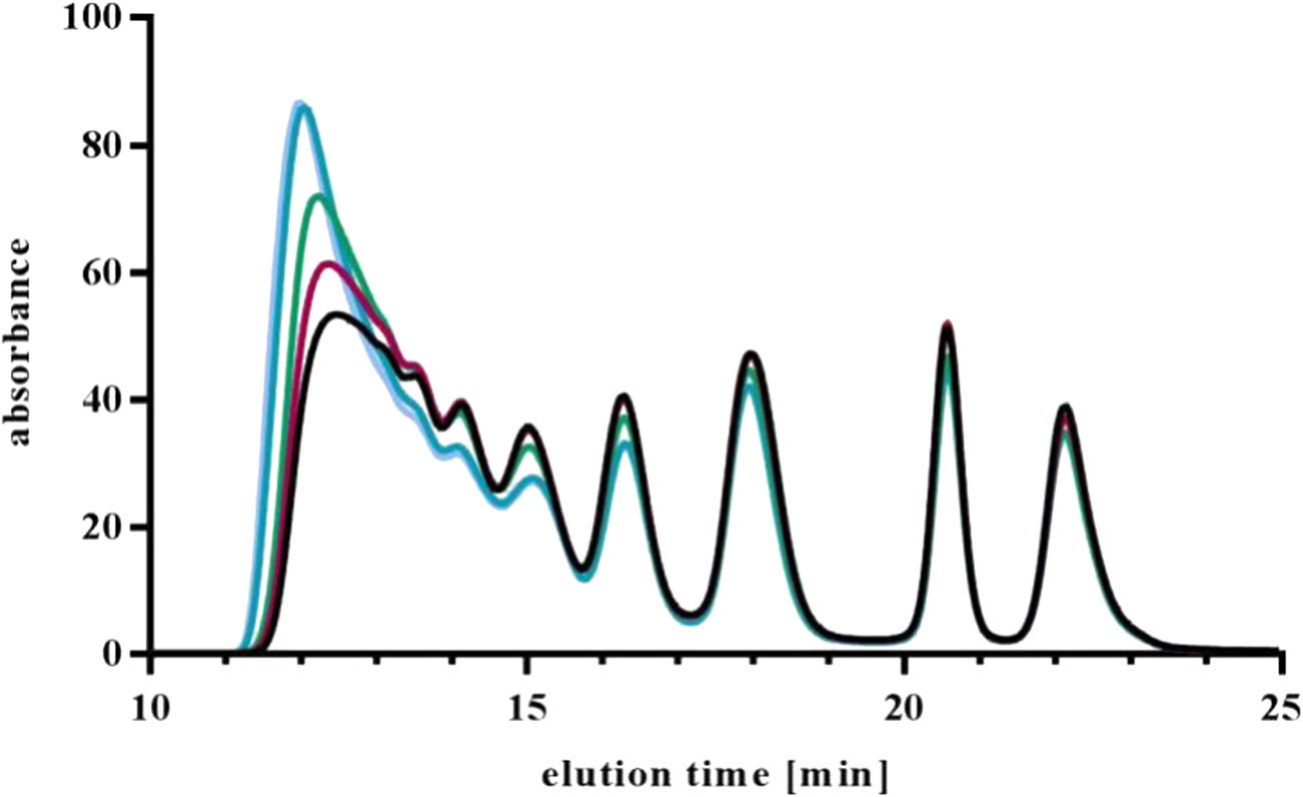
Reproducibility of IC generation. ICs of the drug and pAb <CDR> ADA were generated multiple times and IC patterns were monitored by UV-SEC. The CV of the area under the curve was less than 5%.

Reproducibility of defined ICs was tested by multiple, independent generation of ICs of drug and pAb <CDR> ADA of same concentration and ratio and analysis by SEC. No differences in IC pattern and a CV of 5% verified the robustness of IC generation (Fig. 10).

### Bioanalytical Method Development: Total Drug Quantification by Acid Dissociation ELISA

**Fig. 11:**
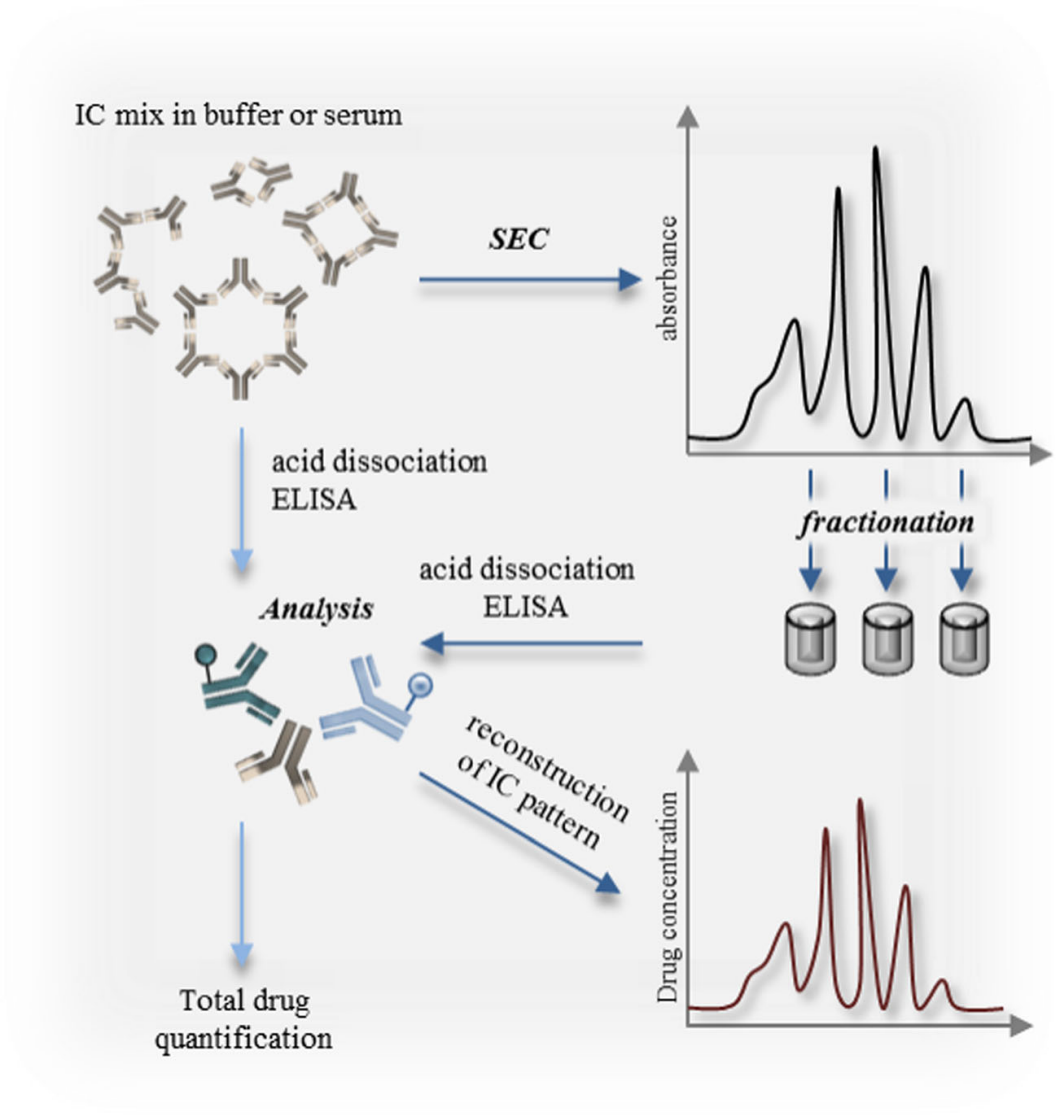
Schematic overview of the total drug quantification via acid dissociation ELISA and the reconstruction of the IC pattern via SEC and acid dissociation ELISA.

To quantify total drug, i.e. free drug and drug entirely complexed with ADAs and in the presence of ADA excess, an acid dissociation ELISA was developed as standard bioanalytical method (Fig. 11).

Drug and < CDR> ADA (mono- or polyclonal) were mixed in a ratio of 1 to 1.5 in buffer or serum for at least 1 h. Dissociation of formed ICs was forced by pH adjustment to pH 2, followed by the addition of excess capture and detection reagents and subsequent neutralization. Complexes of drug, capture and detection Abs (+ ADAs) were transferred to a streptavidin-coated 96-well microtiter plate. The read-out was performed with a Fab-HRP conjugate directed against Dig with HPPA as HRP substrate (16). The calibration was performed with monomeric, non-complexed drug. QCs with monomeric drug spanning the whole calibration range (high concentration QC (H-QC), mid-concentration QC (M-QC), and low concentration QC (L-QC)) were used to guarantee technical accuracy and precision (Table III). Furthermore, the performance and validity of the assay were verified with IC-QCs, i.e. ICs with defined concentrations of drug and ADA (high, mid, and low concentrated IC-QCs, Table III). All QCs are in line with the U.S. food and drug administration (FDA) guideline for bioanalytical method validation (17). The described assay method showed sufficient ADA tolerance for correct drug quantification and a sensitivity of less than 1 ng/ml sample concentration.

**Table III:** Accuracy and precision of QCs and IC-QCs for acid dissociation ELISA

	Drug + pAb <CDR> ADA *n* = 15		Drug + mAb <CDR> ADA *n* = 8		Drug + < H-Fc > ADA *n* = 9	
Sample	Accuracy [%]	Precision [% CV]	Accuracy [%]	Precision [% CV]	Accuracy [%]	Precision [% CV]
H-QC	92	8	94	14	91	13
M-QC	105	7	107	6	93	15
L-QC	91	5	96	6	98	13
H-IC-QC	83	10	120	5	100	15
M-IC-QC	89	7	118	5	99	18
L-IC-QC	101	8	104	7	93	13

H-QC: 300 ng/ml drug; M-QC: 150 ng/ml drug; L-QC: 7.5 ng/ml drug; H-IC-QC: 300 ng/ml drug +450 ng/ml ADA; M-IC-QC: 150 ng/ml drug +225 ng/ml ADA; L-IC-QC: 7.5 ng/ml drug +11.25 ng/ml ADA)

This approach was adjusted to ICs of drug and < H-Fc > ADAs by using a different capture and detection Ab set directed against the CDR of the drug (Table III, right column).

### Drug Quantification in SEC Fractions and Immune Complex Pattern Reconstruction

Besides total drug quantification, reconstruction of the IC pattern in serum should enable dedicated *in vivo* studies and analysis. ICs of drug and ADA were incubated in 100% serum for at least 1 h and subsequently separated via SEC. Fractions were collected and transferred to the previously described acid dissociation ELISA. The concentration of the drug was determined for every collected fraction. Combining the elution times of the fractions with determined drug concentrations, the IC pattern in serum could be reconstructed (Fig. 11).

**Fig. 12:**
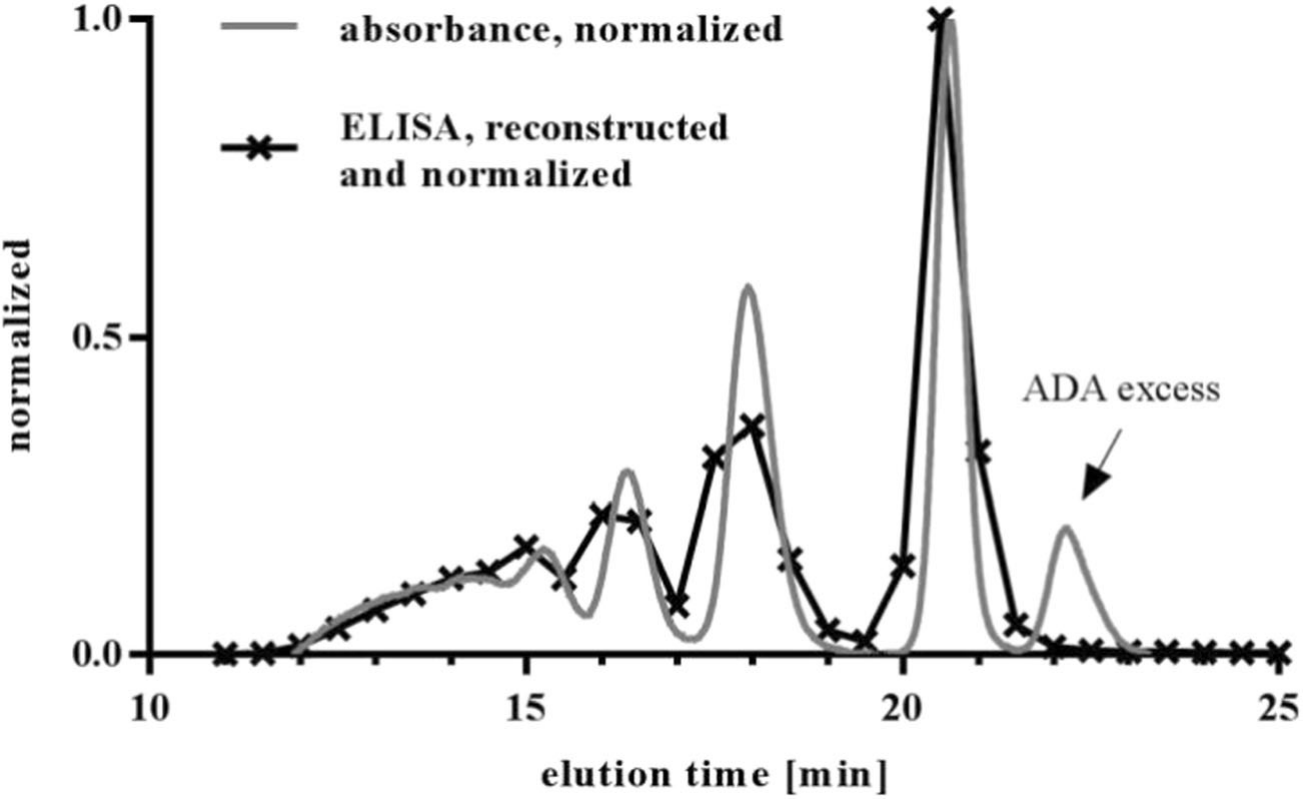
Reconstruction of IC pattern via SEC and acid dissociation ELISA. An IC mixture of 100 μg/ml drug and 150 μg/ml pAb <CDR> ADA was generated and separated by SEC. The drug concentration in collected fractions was determined by acid dissociation ELISA. The graph illustrates the overlay of the UV-SEC chromatogram of the separated IC mix and reconstructed IC pattern generated by ELISA.

Figure 12 illustrates the reconstitution of an IC of drug and pAb <CDR> ADA in serum with the upper described method set-up of SEC and acid dissociation ELISA. Comparison of reconstructed IC pattern with an IC generated in buffer and visualized at 280 nm showed no differences in the IC distribution aside from the ADA excess peak around 22.5 min. As the acid dissociation ELISA only measured drug, this peak was not visible in the reconstructed IC pattern.

To exclude loss of ICs due to the selected SEC method or during the acid dissociation process, the mass balance of injected and recovered ICs (from collected SEC fractions) was assured. The recovery of the injected drug fully complexed with ADAs was in the tolerance range of ±20% (Table IV).

**Table IV:** Mass balance of ICs separated by SEC and quantified by acid dissociation ELISA. Recovery of drug from serum and buffer samples

Sample in:	Drug + pAb <CDR> ADA	Drug + mAb <CDR> ADA	Drug + mAb < H-Fc > ADA
serum	93%	109%	85%
buffer	107%	–	91%

## Discussion

As IgG is the main Ab class used for therapeutic treatment, a human non-binding monoclonal IgG_1_ (PGLALA) was used as drug surrogate. The PGLALA mutation can be found in several drug candidates (8–10, 18–20). The drug is a non-binder excluding target mediated drug disposition (TMDD) and ensure exclusively IC-mediated effects in biological matrices (like serum) or in potential, future *in vivo* studies.

Several investigations have shown that immune responses against therapeutic Abs in human lead to the formation of ADAs which are directed against the CDR of the drug (2;4). We chose a pAb against the CDR to best represent the natural, polyclonal immune response and in accordance with the recommendation of the FDA with regard to ADA positive controls used for immunogenicity testing (21).

To study the influence of different epitopes and ADA properties on IC formation and bioanalysis, two further ADA surrogates were chosen; a mAb <CDR> ADA and a mAb < H-Fc > ADA (Fig. 1). Although binding to epitopes in the same region, potential differences between ICs with mAb and pAb <CDR> ADAs and the influence of clonality were tested. ICs of drug and mAb < H-Fc > ADAs were investigated to reflect conditions which could be found in pre-clinical studies in animals, where an immune response against the human Fc domain is possible (4) and give hints on potential epitope effects on IC formation and IC properties.

The standard method for visualization of IC formation was SEC with online UV detection. To allow MW determination of generated IC species by SEC, the column was calibrated using a commercially available SEC protein standard mix. We could show that the MW evaluation including or excluding the standard mix component uracil had dramatic influence on the recalculated values of generated ICs (Fig. 3c). Calibration without uracil led to a much more accurate approximation to the actual MW of the IC species (determined by SEC-MALS, AUC, and NS-TEM). As uracil is a small molecule, it hardly represents the elution behavior of Ab-ICs and therefore led to overestimation of MWs. In general, the protein standard should be chosen with caution. At best, standard components should be very similar in size and structure with the molecules to be analyzed. Furthermore, SEC separates molecules on the basis of their hydrodynamic radius, rather than their MW. Therefore, determination of MW via SEC protein standards can be misleading and should be verified with orthogonal methods. We decided to determine the actual MW of generated ICs via SEC-MALS, AUC, and NS-TEM to overcome the limitations of SEC and to perform an inter-method calibration of the SEC for following experiments.

For SEC-MALS analysis, the MALS detector was connected to a SEC and therefore the results were highly dependent on the resolution capability of the used SEC column. Separation of the IC mixture generated by drug and pAb <CDR> ADA resulted in five defined peaks which were identified by the SEC-MALS analysis as monomeric excess ADA and ICs of dimers, tetramers, hexamers, and octamers. Resolution of HMW complexes was not possible under applied chromatographic conditions (Table I). Comparison of the MW values of separated IC species determined by SEC-MALS or by SEC protein standard calibration was performed, whereby SEC-MALS indicated lower MW values (Fig. 3c). Additional measurements of ICs with mAb <CDR> ADAs showed that ICs with sizes corresponding to tetramers, hexamers, and octamers were found. The absence of a detectable dimer can be due to chosen conditions, like concentration of drug and ADA. Although the ICs of drug and mAb < H-Fc > ADA showed a different SEC pattern (Fig. 2), SEC-MALS data identified dimeric and tetrameric ICs as main species, as for the other studied complexes (Table I).

AUC of separated ICs should further confirm MW data from SEC-MALS. IC species of drug and pAb <CDR> ADA were separated and fractionated via SEC. The resulting isolated IC species were analyzed via AUC. Found MWs were in line with SEC MALS data (Table II). Besides the mass determination, AUC allowed further statements about the stability of generated IC species. The whole procedure of sample preparation and measurement included dilution, pooling, concentration, freezing, and thawing as well as centrifugation. Figure 4 illustrates the superposition of sedimentation coefficients and demonstrates that the separated species were still the main component, especially in the dimer fraction. The tetramer and hexamer fractions showed further, mainly larger species (oligomers and aggregates) but scarcely smaller protein species (i.e. monomers). This indicates that generated ICs were highly stable even under extensive sample treatment conditions.

Besides SEC-MALS and AUC, NS-TEM was used as an additional method to determine MW and structural information of the ICs. Similar to AUC, ICs of drug and pAb <CDR> ADA were generated and separated via SEC. The defined IC species were visualized via NS-TEM confirming the findings of SEC-MALS and AUC (Fig. 5). Although sample preparation was very extensive, expected IC species (namely, dimers and tetramers) were found in high numbers. Besides MW and stability determination, NS-TEM provided insights into the structure of generated ICs. Cyclic ICs were found as predominant arrangements. Furthermore, tetramers showed heterogeneity in the cyclic structure with Fc regions facing inwards or outwards of the ring structure, presumably due to the polyclonal nature of the ADAs (Fig. 5c). Tetramers of drug and mAb <CDR> ADA did not show this phenomenon, but rather a homogeneous pool of cyclic tetramers with the Fc region facing outwards of the ring (Fig. 5b). Johansson et al. performed electron microscopy with ICs of mAb TS1 and its anti-idiotypic mAb (αTS1). In contrast to our approach they analyzed the total IC mixture, however tetrameric, hexameric, and octameric ring structures were found as predominant structures. Similar to our findings, no dimeric complexes were found for the monoclonal anti-idiotype (22).

Reproducible generation of defined ICs is mandatory for valid results and analysis. Different aspects such as reproducibility, dilution and temperature stability, influence of drug to ADA ratio, concentration, epitopes, and ADA properties were tested. We demonstrated that multiple, independent mixing of ICs under the same conditions led to the same UV-SEC patterns (Fig. 10) and that generated IC species are highly stable towards increased temperature and dilution (Figs. 8 and 9).

A drug to ADA ratio of 1 to 1.5 was confirmed as ideal to fulfill the requirements of total complexation of drug with ADAs and little ADA excess. With regard to potential *in vivo* studies with preformed ICs, excess monomeric drug could lead to misinterpretation of PK data by a total drug assay and therefore should be avoided.

ADA properties, like clonality or specificity also had influence on IC formation. The polyclonality of the pAb <CDR> ADA surrogate led to formation of a dimeric complex which was missing in the case of mAb <CDR> ADAs although both ADAs bind to the CDR of the drug. Besides potential steric aspects (see NS-TEM) chosen concentrations or ratios of drug and mAb <CDR> ADA could also have influence. The ADA surrogate directed against the Fc region of the drug formed ICs which had similar MWs (see SEC-MALS data) but a different UV-SEC pattern (Fig. 2), probably due to different molecular structures.

Quantification of total drug, free and fully complexed with ADAs, is a requirement for analysis of future *in vivo* studies (total drug PK) (23). Rat serum was used as biological matrix to be prepared for planned *in vivo* studies in rats. An acid dissociation ELISA was developed as standard bioanalytical assay to perform total drug quantification/PK determination eliminating potential ADA interference. Calibration was performed with monomeric drug and QCs all over the calibration range guaranteed technical accuracy. The complete dissociation of the ICs and capturing of the drug was monitored with IC-QCs. The presence of serum had no effect on drug quantification, as no differences to buffer samples were observed. Experiments indicated a sufficient ADA tolerance (1.5-fold higher ADA concentration than drug concentration) of the established acid dissociation ELISA as shown by accuracy and precision data well within in the tolerance range requested for PK analysis (Table III) (17).

Besides the quantification of the fully-complexed and free drug in serum (PK determination), reconstruction of the IC pattern in serum to monitor potential changes over time is of high interest. Different research groups developed technologies and methods for the analysis and quantification of ICs in buffer and sera (6,24–26). Boysen et al. developed a SEC-based method to visualize ICs in sera by adding Fab fragments with a fluorescence-tag to preformed ICs (27). Although a fast and generic method, it has different drawbacks: with a limit of detection of 10 μg/ml, only high concentrated samples can be studied. Furthermore, binding of the Fab fragments to the ICs leads to a shift in size. As the number of bound Fab fragments rises with increasing IC size conclusions about size and concentration are challenging. Additionally, multiple binding of Fab fragments can lead to a shift of ICs into the low-resolution part of SEC columns. Regenass-Lechner et al. (28) used a combination of SEC and specific ADA and IC ELISA to identify ICs in cynomolgus monkey serum and built a methodical corner stone for this project.

Accurate monitoring of changes in the IC pattern *in vivo* opens new opportunities to study the impact of ADA generation and subsequent IC formation on drug PK.

Online UV detection of ICs in serum is not feasible, as the serum protein-dependent signal background cover the specific ICs detection. A combination of SEC for MW determination and acid dissociation ELISA for analyte specific detection was established to overcome this issue. As illustrated in Fig. 11, serum samples containing ICs were separated via SEC and the drug concentration in the collected fractions was determined with a drug-specific acid dissociation ELISA. Plotting the elution volume of the fraction against the drug concentration allowed the reconstruction of the IC pattern.

*In vivo* studies with preformed and defined ICs require a valid bioanalysis excluding any method-dependent bias of the results. The comparison of the IC pattern generated in buffer and detected online at 280 nm with the reconstructed IC pattern generated in serum (Fig. 12) showed no differences in the profile. It can be concluded that serum components did not influence IC formation. Furthermore, the separation of the ICs in serum by SEC is not affected and the process of SEC did not lead to loss of ICs in the HPLC system through aggregation or adherence to filters. This was verified by comparing the amount of injected drug with the amount of drug quantified in collected fractions. A drug recovery of ±20% was accepted as valid result (Table IV).

## Conclusion

The methods for generation and characterization of ICs, in addition to quantification of drug entirely complexed with ADAs and the reconstruction of IC profiles in serum were established to enable dedicated *in vivo* studies. *In vivo* studies with preformed ICs will allow a better understanding of IC clearance and therefore of ADA induced effects on drug PK.

Generated ICs of drug and pAb <CDR> ADA were analyzed via UV-SEC and showed a reproducible IC pattern. The SEC column was calibrated with a protein SEC standard mix to determine the MW of generated IC species. It was demonstrated that calibration via protein SEC standard mix led to highly biased results. Therefore, it is necessary to determine the MW of IC species with further, better suitable methods like SEC-MALS, AUC, or NS-TEM (Fig. 3c, Fig. 5, Tables I and II). Furthermore, SEC columns currently on market are limited in their ability to resolve HMW species and therefore restrict further detailed characterization.

The quantification of drug fully complexed with ADAs was performed with an acid dissociation ELISA and will allow quantitative bioanalysis of future drug PK studies with preformed ICs. This ELISA approach showed a very high ADA tolerance even for high affinity ADA surrogate molecules which form stable ICs.

The reconstruction of IC patterns in serum by combining SEC and acid dissociation ELISA allowed the visualization of IC profile changes without any modifications of the analyte, for example fluorescence tags. The method set-up was optimized for full recovery of ICs from SEC, i.e. the HPLC system for the generation of unbiased IC profiles. Furthermore, the presented bioanalytical concept is highly flexible and can be adapted to a variety of analytes and problems.

The described combination of SEC and acid dissociation ELISA was fully qualified and characterized enabling robust and fast analysis of serum samples obtained from *in vivo* studies with preformed ICs and other approaches.

## Abbreviations

Ab: Antibody
ADA: Anti-drug antibody
AUC: Analytical ultracentrifugation
Bi: Biotin
CDR: Complementary determining region
CV: Coefficient of variation
Dig: Digoxigenin
ELISA: Enzyme-linked immunosorbent assay
FcγR: Fc gamma receptor
HMW: High molecular weight
IC: Immune complex
IgG: Immunoglobulin G
MALS: Multiple angle light scattering
NS-TEM: Negative staining transmission electron microscopy
PK: Pharmacokinetics
QC: Quality control
SEC: Size exclusion chromatography
UV: Ultra violet
